# Comparing Meal Satisfaction Based on Different Types of Tableware: An Experimental Study of Japanese Cuisine Culture

**DOI:** 10.3390/foods10071546

**Published:** 2021-07-04

**Authors:** Tomoko Hasegawa, Nobuyuki Sakai

**Affiliations:** 1 Faculty of Psychology and Sociology, Taisho University, 3-20-1 Nishi-sugamo, Toshima-ku, Tokyo 177-8470, Japan; 2 Graduate School of Arts & Letters, Tohoku University, 27-1 Kawauchi, Aoba-ku, Sendai 980-8756, Japan; nob_sakai@tohoku.ac.jp

**Keywords:** plastic tableware, natural tableware, timing of meal, satisfaction, ready-made meal, Japanese cuisine culture

## Abstract

In Japan, as in other countries, the externalization of food preparation is increasing. Japanese people are interested in the combination of food and tableware and they are concerned about transferring ready-made meals from plastic containers to natural tableware. This study aimed to examine the varying evaluations of meals due to differences in tableware. In this study, we investigated the effect of tableware on meal satisfaction, which is emphasized in Japanese culture. We studied the difference in the evaluation of ready-made meals (a rice ball, salad, croquette, and corn soup) before, during, and after a meal under two conditions: plastic tableware and natural wooden tableware. The results showed that there was no difference in the perceptual evaluation of taste and texture during the meal, except for the color of the salad and the temperature of the soup. On the other hand, meals served on natural wooden tableware were rated more positively than those served on plastic tableware before and after meals. These results suggest that, in Japan, the use of tableware, even for ready-made meals, increases the level of meal satisfaction. These findings have implications for both the providers and consumers of ready-made meals as well as the food industry.

## 1. Introduction

Tableware is an indispensable component of the eating experience. Many studies on food acceptance have examined the relationship between food and tableware [1,2,3,4,5]. In a review of 67 studies where tableware was considered as a variable, Jinbo and Imoto [6] compared the research methods in studies undertaken in Japan (26 studies) and other countries (41 studies) by dividing their research objectives into the quantity and quality of food and meals. In studies of countries other than Japan, 33 out of 41 studies focused on the quantity of food and meals, but only eight studies focused on quality, with most of the quality studies focusing on taste (perceived taste, flavor, saltiness, sweetness, etc.) and preference. On the other hand, in Japanese research, 25 of the 26 studies focused on the quality of food and meals. These studies focused on many perspectives not seen in the studies of other countries, such as good appearance, compatibility of the tableware with the food, and the comfort of the meals in addition to appetite and deliciousness.

These differences in the perspectives of the Japanese studies compared to the other countries’ studies on tableware reflect two different attitudes. First, the recent focus on healthy eating. In Europe and the United States, healthy eating and the control of food intake are considered the most important issues because of the high prevalence of obesity [7]. However, in Japan, where obesity is considered an issue, the actual percentage of overweight and obese people is the lowest among OECD countries [7]. Second, differences in the cultural background of cuisine. Comparing the different styles of cuisine in representative countries from around the world, French cuisine is said to be aromatic, Chinese cuisine is tasty, and Japanese cuisine is a feast for the eyes [8]. This feast is in part due to Japan’s food culture, in which tableware plays an important role during meals. Kaiseki cuisine, one of the most refined styles of Japanese cuisine that dates back to the 16th century, reflects the importance of tableware and the arrangement of the food on the tableware. When selecting tableware, one considers the color, tone, shape, harmony with the food, and season [6]. Because of this cultural background, there is an unparalleled variety of Japanese tableware [9]. Historically, although the meals of the masses were simple [10], since the 1970s, one traditional Japanese meal style, Ichiju-sansai, translated as “a soup and three dishes”, has been the basis of menu planning [11]. In Ichiju-sansai, each item is served on a separate dish for each diner and, therefore, a variety of tableware is found in each home. In Japan, a range of practical books have been published by culinary experts with tips on how to select tableware and serve food [12], which are regularly used as references for enjoying the preparation of everyday meals and increasing meal satisfaction.

As in Europe and the United States [13,14,15,16], “the externalization of food preparation” has been increasing in Japan [17,18,19,20]. This is defined as the dependence on food preparation and consumption outside the home and includes both ready-made meals as well as the catering trade [20]. This externalization is mainly due to the growing availability and convenience of ready-made meals. In the Japanese food market, ready-made meals/food (i.e., meals/food that can be eaten without cooking or heating at homes, workplaces, or schools, such as lunch boxes and daily dishes with short shelf lives [21]) were valued at JPY 10.3 trillion in 2020 (14.2% of the total market), showing an increase of 27.3% over the past 10 years [22]. The household food expenditure percentages of eating out and consuming ready-made meals by age group shows that eating out is less common for older people, while ready-made meals made up 13% of food expenditure for all age groups in 2019 [23]. These results indicate that ready-made meals have become popular with all generations.

In Japan, especially among women, there have been conflicting attitudes regarding the use of ready-made meals and tableware. Rapid economic growth since the 1960s has led to an increase in the population of unemployed housewives [24]. At that time, cooking programs on TV and cooking magazines started to appear, and housewives were encouraged to prepare home-cooked meals with love for their families [11]. Housewives used ready-made meals, but their use was accompanied by a sense of guilt. Therefore, when using ready-made meals, it is natural to transfer the food to personal dishes. During the same period and much earlier, people in Europe and the United States might have also regarded women’s use of ready-made meals as “the low moral status of convenience-orientation in food preparation” [25], however, it did not encourage many women to transfer ready-made meals to plates.

However, with the increase in the number of ready-made meals over the past 20 years, people, including housewives, still use plastic containers that come with the ready-made meal rather than transferring food to personal cutlery, or use cooking pans directly as tableware [17,26,27]. The issue of transferring ready-made meals from plastic containers to personal crockery is still of great interest to the general public and is a major topic of debate, as seen on social networking services and the mass media. The increase in the availability of ready-made meals in Japan is similar to that in other countries, but the above cultural background is the reason why there is such a debate on whether ready-made meals should be transferred to personal containers.

In Japan, the food culture described above is still changing and questions regarding the kind of effect tableware has on the mental satisfaction of daily eating have become of interest. The purpose of this study was to examine the differences in the visual impression before a meal, the perceptual evaluation during a meal, and an after-meal evaluation when using plastic tableware versus natural tableware.

This experimental study highlights two features of interest regarding food and tableware. The first is the timing of the assessment of the food and the makeup of a meal. Human eating behavior changes over time—before, during, and after meals—due to different aspects such as pleasure and hunger level [28]. However, in previous studies on food and tableware, most evaluations were based on the appearance of the food at a single point in time without participants actually tasting it [5]. Only a few studies examined the changes in assessments before and after eating a meal [29,30,31,32]. In addition, in previous research when tasting the food, there was only one type of food (including beverages) or one plate provided, and the assessment of the food tended to be based on only a few basic variables such as taste, liking, palatability, and healthiness [1,29,30,33]. In contrast, this study uses a very different perspective than the previous studies by providing meals similar to people’s typical eating habits. Specifically, the meals consisted of multiple dishes as in a typical meal and the evaluations of the meals occurred at three time points (before, during, and after the meal), thus enabling us to compare the changes in the evaluations before and after the meal and the differences in the types of tableware. The second feature of interest was the focus on the background of ready-made meals and the culture of cuisine in the study of tableware. Previous studies, such as Laguna et al. [33], have focused on ready-made meals; however, this study is the first to focus on a country-specific cuisine culture with respect to food and tableware. Although previous studies in Japan have considered tableware from the perspective of the cuisine culture, little consideration has been given to the rapidly increasing use of ready-made meals. Considering these points, this study provides new insights into meal satisfaction in the case of ready-made meals; the findings are applicable to not only ready-made meal consumers but also the food industry.

## 2. Materials and Methods

### 2.1. Participants

Twenty students (10 males and 10 females) from a private university in Tokyo participated in the experiment. The mean age was 20.70 years (*SD* = 1.75), and the mean body mass index (BMI: weight (kg)/height (m)^2^) was 22.39 (*SD* = 4.78) for men and 21.19 (*SD* = 2.14) for women. 

### 2.2. Procedure

The participants were instructed not to eat or drink for 3 h before the experiment. They then ate the pre-set meal as lunch in the laboratory. All participants ate the same meal twice: once using plastic tableware and once using natural wooden tableware, with an interval of one week. The meal was evaluated at three time points: before, during, and after the meal. Before the meal, the participants evaluated it according to its overall appearance. During the meal, the participants were instructed to take four or five bites of each item, taste them carefully, and then evaluate the meal. After they finished eating, the participants gave an overall impression of the meal. The order in which the participants were assigned to the two conditions was counterbalanced. The timings of the meals were measured in seconds from the beginning to the end of the meal. 

### 2.3. Materials

#### 2.3.1. Foods

The meal comprised four identical items of food in both plastic and natural tableware.

The items included salted rice balls (shio-nigiri), salad (namayasai-sarada wafu-doreshingu: radish, lettuce, red and yellow bell peppers, and purple cabbage) with a Japanese dressing, croquettes (korokke), corn soup with kernels (tsubuiri-kohn-supu), and Japanese green tea (ryokucha).

#### 2.3.2. Tableware

**Figure 1:**
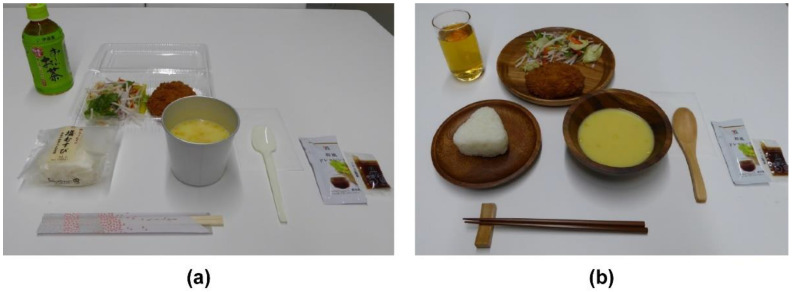
The meals used for each experimental condition. (**a**) Plastic tableware condition; (**b**) natural wooden tableware condition.

In the plastic tableware condition, the salted rice balls were in their plastic wrappers, the salad and croquettes were in a plastic pack, the corn soup was in a paper soup cup with high heat retention, and the tea was in a PET bottle (350 mL). Utensils included disposable chopsticks and plastic spoons. In the natural wooden tableware condition, the salted rice balls, salad and croquette, and corn soup were served separately on wooden plates, the tea was served in a glass, and the utensils included wooden chopsticks on a chopstick rest, and wooden spoons (Figure 1).

### 2.4. Evaluation Criteria

Participants were asked to rate the meal at three time points: before, during, and after the meal. Before the meal, the overall appearance of the food was evaluated according to 6 criteria: “full-hungry”, “looks unpalatable-looks tasty”, “frugal-gorgeous”, “bland-heart-warming”, “unhealthy-healthy”, “ordinary-exclusive”. Eight criteria were evaluated during the meal. Each of the 4 food items was evaluated on two different criteria: rice ball (taste) “not salty-salty”, (texture) “loose-compact”; salad (texture) “limp-crunchy”, (color) “pallid-colorful”; croquette batter (texture) “crispy-soggy”, croquette ingredients (texture) “moist-dry”; corn soup (heat) “tepid-hot”, (taste) “cannot taste the natural sweetness of corn–can taste the natural sweetness of corn”. For the after-meal survey, 8 criteria were evaluated: “it was unpalatable-it was tasty”, “full-hungry”, “disliked-liked”, “discontented-contented”, “unhealthy-healthy”, “do not want to eat it again-want to eat it again”, “uneasy-relaxed”, “was unfamiliar-was familiar”. The rating was based on a 9-point Likert scale, with the 2nd and 8th points of each criterion written as anchors. The reason why an anchor in the middle category 5 was not included was because Japanese have a tendency not to use extreme ratings to avoid conflict [34]. By setting anchors at 2 and 8, we made it easier for participants to use the answers at 1 and 9 if desired.

### 2.5. Ethics Approval and Consent to Participate

Ethical approval was obtained from the relevant authorities and informed consent was obtained from each participant. The participants were informed of the purpose of the study, that participation would be voluntary, that the freedom to discontinue participation would be respected, that the responsibilities of the experimenter and participant would be clarified, that equal consent would be established, and that personal information would be protected. All research was performed in accordance with the Declaration of Helsinki. 

### 2.6. Statistical Analysis

Two-way repeated measures ANOVAs by condition (plastic tableware/natural wooden tableware) and timing (before the meal/after the meal) were conducted to examine the changes according to time for the three near-identical criteria that were rated before the meal and after the meal (hungry, healthy, and looks tasty). When the main effect was significant, multiple comparisons were performed. For the remaining criteria (before, during, and after the meal) and meal timings, *t*-tests were conducted between the two conditions of plastic and natural wooden tableware. Effect sizes were calculated using both ANOVA (*η*^2^) and *t*-tests (*r*). The significance level was set at 0.05, for all criteria. However, due to the small number of subjects in this study (*n* = 20), those with large effect sizes but marginal significance levels of less than 0.10 were still included.

Data analysis was performed using SAS ver9.4 (SAS Institute, Inc., Cary, NC, USA).

## 3. Results

First, two-way ANOVAs were conducted on three criteria: hunger, taste, and healthiness (Table 1). The results showed that the main effect of time was significant for “hungry” (*F* (1, 19) = 377.19, *p* < 0.001, *η*^2^ = 0.952), and hungry was stronger before meals than after meals. For “tasty”, the main condition effects (*F* (1, 19) = 7.84, *p* < 0.05, *η*^2^ = 0.292) and timing (*F* (1, 19) = 16.48, *p* < 0.01, *η*^2^ = 0.465) were significant, with the natural wooden tableware condition being rated as tastier than the plastic one and after the meal it was perceived as tastier than before the meal. For “healthiness”, the main condition effect was significant (*F* (1, 19) = 30.10, *p* < 0.001, *η*^2^ = 0.613), with the natural wooden tableware condition being rated healthier than the plastic one. Additionally, the following factors related to the main effect and the interaction between condition and timing were found to have a marginal significance level of less than 0.10. The interaction of the variable “tasty” (*F* (1, 19) = 3.22, *p* < 0.10, *η*^2^ = 0.149) was examined for a simple main condition effect. The results showed that the participants tended to rate the meal as tastier after using the plastic tableware (*t* (19) = 3.45, *p* < 0.01, *r* = 0.620), whereas there was no significant difference between the before and after meal scores for the natural wooden tableware. The main effect of time on “healthy” tended to be significant (*F* (1, 19) = 3.22, *p* < 0.10, *η*^2^ = 0.149), with the participants tending to rate the meal as healthier after meals than before.

Next, *t*-tests between the two conditions were conducted to examine the differences in the evaluation criteria (Table 2). In the before the meal evaluation, the natural wooden condition was rated as more “gorgeous” (*t* (19) = 4.08, *p* = < 0.001, *r* = 0.68), “heart-warming” (*t* (19) = 6.33, *p* = < 0.001, *r* = 0.82), and “exclusive” (*t* (19) = 3.75, *p* = < 0.01, *r* = 0.65) than the plastic tableware condition. During the meal evaluation, compared to the plastic tableware condition, the natural wooden tableware condition was more “colorful” (*t* (19) = 2.85, *p* = < 0.05, *r* = 0.55) for salads, while the soup was less “hot” (*t* (19) = 2.47, *p* = < 0.05, *r* = 0.49). No significant differences were observed in the other item criteria. In the after-meal evaluation, compared to the plastic tableware condition, the natural wooden tableware condition was rated higher in terms of “liked” (*t* (19) = 2.11, *p* = < 0.05, *r* = 0.44), “contented” (*t* (19) = 3.15, *p* = < 0.01, *r* = 0.59), “want to eat it again” (*t* (19) = 2.97, *p* = < 0.01, *r* = 0.56), and “relaxed” (*t* (19) = 2.82, *p* = < 0.05, *r* = 0.54). The means for the timings of the meals were 913.40 *S* (*SD* = 214.92) for the plastic and 882.35 *S* (*SD* = 212.24) for natural wooden tableware conditions, indicating no significant difference between the two conditions (*t* (19) = 1.19, *p* = 0.247, *r* = 0.26).

## 4. Discussion

In this study, we examined whether there were any differences in people’s evaluations of a meal before, during, and after the meal depending on the different tableware used (plastic vs. natural wooden). Overall, the results showed that the evaluations both before and after the meal were more positive for the natural wooden tableware than the plastic tableware. No differences between the plastic and natural wooden tableware were noticed for hunger (before and after the meal), the perception of taste and texture of the food (during the meal), and in how long it took to finish the meal.

There was no difference in the perceptual evaluation of each item of food during the meal depending on the type of tableware, except for the color of the salad and the heat of the corn soup. With regard to the color of the salad, it is suggested that the color balance between the wooden container and the salad is more vivid than that between the plastic container and the salad because of the distinct color contrast of the wooden container. For the heat from the corn soup, in the plastic condition, participants used paper cups with high heat retention properties while in the natural wooden condition, participants used a shallow wooden container with a large surface area. Therefore, even though the temperature of the corn soup was the same in both conditions at the time of serving, it was probably lower for the natural condition at the time of consumption and evaluation. There was no effect of tableware on other perceptions such as taste and texture.

On the other hand, both before and after the meal, positive evaluations were observed for the meal using natural wooden tableware. This suggests that the visual aesthetics of the meal had a great influence on its evaluation before meals. The visual information implies that the food on the plastic tableware is a ready-made meal while the food on the natural wooden tableware is a homemade meal. In general, people hold negative beliefs about ready-made meals in terms of their taste, nutritional value, and healthiness [35,36]. In this study, it was suggested that the expectations when eating a meal with plastic tableware were lower than those when using natural wooden tableware.

The multiple aspects of human eating are intimately interrelated through the phases of before, during, and after the meal. The multilevel model of food intake over time [28] shows the changes in each of the seven aspects: (i) pleasure [37,38], (ii) hunger level, (iii) satiation/satiety cascade signals [39,40,41,42,43], (iv) origin of signals and signal carriers, (v) brain processes, (vi) behavioral changes including those in the digestive system, and (vii) general modulatory factors, and the interactions among the aspects over time before, during, and after the meal. Thus, human eating is complex and changes over time.

However, in this study, although there were no differences in the sensory intensity of taste, color, and texture, regardless of the type of tableware, the effects of the tableware condition before the meal continued after the meal, and the natural tableware condition had a more positive effect than the plastic tableware condition at both times. This might be due to the influence of factors that are outside the field of view of the multilevel model of food intake over time; that is, within a certain cultural context, the visual information gained before eating a meal affects people’s cognition. A belief that Japanese people share with those from other countries is that eating with natural tableware evokes the image of homemade food, which is healthier and thus is more positively perceived [35,36]. Additionally, in Japan, homemade meals prepared by housewives are a sign of love for the family. Therefore, the belief that it is natural to transfer ready-made food from plastic containers to plates [11] has had a long-lasting influence.

On the other hand, in this study, the evaluation of food during the meal did not affect the after-meal evaluation. In the plastic tableware condition, the ratings of taste were higher after the meal than before the meal. The following factors are suggested to have contributed to this change, because the meals served on the plastic tableware were clearly intended to be seen as ready-made meals. The participants predicted that the meal would not have a strong flavor and would be greasy, as this is the image that most Japanese people have of ready-made meals. However, when the participants actually ate the meals, they found that this was not the case, and the meals were perceived to be unexpectedly tasty. Moreover, in both tableware conditions, the meal tended to be rated as healthier after the meal than before. In a study by Laguna et al. [33], five types of ready-made meals (pasta, meatballs, salad, beans stew, and a sandwich) were compared under three conditions: (i) “meal-photo condition” where photographs of the meal on a plate were shown, (ii) “pack-alone condition” where only the packaging of the food was shown, and (iii) “tasting + pack condition” where the meal was tasted and the packaging was presented beside the meal. The meals were rated using three criteria: liking, satiety, and perception of healthiness. The results showed that, for salad and bean stew, liking was significantly lower in the “pack-alone condition” than in the “meal-photo condition”, but there was no difference in liking between the “meal-photo condition” and the “tasting + pack condition”. The same difference between the conditions in the healthiness perception was observed for meatballs, salads, and sandwiches. In contrast, in the present study, the fact that, for the natural tableware condition, the participants did not rate the meal higher in terms of level of on the criteria “tasty” after the meal (than before the meal) might be due to the ceiling effect caused by high “tasty” expectations before the meal. Moreover, the fact that the main effect of timing tended to be an increase in healthiness ratings after the meal compared to before the meal suggests that, regardless of the tableware, the actual eating of the meal led to a more positive evaluation of the meal contents which, in turn, led to the perception of the meal as healthy.

This study’s findings suggest that the use of natural tableware may increase one’s sense of satisfaction after a meal, even for a ready-made meal. The social background of the externalization of food preparation includes (i) socio-demographic shifts such as an increase in the number of single people and the rate of female employment [44]; (ii) an increase in the number of people who have strong time constraints or time scarcity (review [45]), that ready-made meals are not only for people with jobs and families [46,47] but also for young people [48]; (iii) a general decline in cooking skills ([49]; review [50]); and other factors ([51,52], review [53]). Even if it is difficult to prepare a meal, simply transferring food from plastic containers to natural containers could be a simple way to increase meal satisfaction.

## 5. Conclusions

In Japan, visual aesthetics, harmony between food and tableware, and home-cooked meals are traditionally very important. However, there has been a marked increase in the use of ready-made meals/food in the past 20 years. Most of the studies on tableware and food from countries other than Japan focused on healthy eating, while most of the Japanese studies focused on the quality of the meal, such as the harmony between food and tableware. Few Japanese studies considered the use of ready-made meals. In this study, we examined the differences in the assessments of readymade meals at three times-before, during, and after meals—with the meals consisting of multiple dishes in different types of tableware, plastic tableware, and natural wooden tableware. The results showed that there was no difference in the perceptual evaluation of taste and texture during the meal, except for the color of the salad and the temperature of the soup. On the other hand, the meal served with natural wooden tableware was more positively rated than that with plastic tableware before and after the meal. These results suggest that the use of natural tableware, even for ready-made meals, increases the level of meal satisfaction in Japan. This has implications that may encourage further research on the importance of tableware for meals, and may be of interest to a wide range of consumers and the food industry.

This study has certain limitations that could be addressed in further studies. First, since the study only included 20 university students, its sample size was relatively small. The effect sizes of the variables that showed significant differences in the statistical analysis of this study are statistically robust. Therefore, we considered that this was not a major problem. However, to confirm this study’s findings, it may be advisable for future studies to include a larger sample size.

Second, this study did not consider the frequency of consumption of ready-made meals and the use of natural tableware in the daily diets of the participants. In Japan, there are no generational differences in the use of ready-made meals/food [23]. The feelings and attitudes toward the use of ready-made meals as shared in other studies are either ones of guilt about not being able to prepare homemade meals due to time scarcity [36,54] or positive ones [13,14]. Those with positive attitudes toward ready-made meals consumed a higher quantity of ready-made meals and perceived ready-made meals to be healthier and to save time and money [13,14]. The externalization of food preparation not only saves time and effort in preparing meals but also in cleaning up afterward [46]. In the future, it will be necessary to examine how those who consume ready-made meals often and have a positive attitude toward them evaluate ready-made meals served with plastic tableware.

Third, this study only speculates about the influence of Japanese culture on the positive effects of natural tableware. In future studies, it will be necessary to examine the influence of Japanese food culture by conducting international comparative studies and studies in Japan, asking about beliefs regarding transferring dishes when consuming ready-made meals.

Finally, the participants’ evaluations in this experiment before, during, and after the meal were purely based on subjective sensory evaluation. The multi-level model of food intake over time [28] shows that human eating behavior changes with time as the different food/human aspects interact with each other. In future research, physiological indicators, as well as sensory evaluation, should be used to examine whether satiety signals are altered by contextual conditions such as tableware. Moreover, these signals are thought to rise slowly after a meal and peak approximately an hour later. Thus, these may not be fully apparent in assessments immediately after a meal, such as those conducted in this study. Therefore, it is necessary to consider the time factor, as well.

## Figures and Tables

**Table 1:** Descriptive statics and two-way ANOVA results.

		Means				ANOVAs				
		Plastic (*n* = 20)		Natural (*n* = 20)						
Criteria	Timing	*M*	*SD*	*M*	*SD*	Factor	*F* Value	*p*	*η* ^2^	Multiple Comparison
Hungry	Before	7.15	1.04	7.15	0.99	Condition (C)	0.07	0.797	0.004	
	After	2.75	1.02	2.65	0.99	Timing (T)	377.19	<0.001 ***	0.952	before > after
						C × T	0.10	0.761	0.005	
Tasty	Before	5.95	1.43	7.40	1.27	Condition (C)	7.84	0.044 *	0.292	natural > plastic
	After	6.95	1.23	7.60	0.82	Timing (T)	16.48	0.001 **	0.465	after > before
						C × T	3.22	0.084	0.149	
Healthy	Before	5.40	1.27	6.95	0.89	Condition (C)	30.10	<0.001 ***	0.613	natural > plastic
	After	5.90	1.33	7.20	0.83	Timing (T)	3.70	0.069	0.163	
						C × T	0.66	0.425	0.034	

Notes: plastic, plastic tableware condition; natural, natural wooden tableware condition; before, before the meal; after, after the meal. Small, medium, and large effect sizes of *η*^2^ are 0.01, 0.06, and 0.14, respectively, *** *p* < 0.001 ** *p* < 0.01 * *p* < 0.05.

**Table 2:** Descriptive statics and comparison between plastic and natural tableware condition.

	Plastic (*n* = 20)		Natural (*n* = 20)				
Criteria	*M*	*SD*	*M*	*SD*	*t* Value	*p*	*r*
**Before meal**							
Gorgeous	4.35	0.99	5.60	1.39	4.08	<0.001 ***	0.68
Heart-warming	4.15	1.50	6.80	1.54	6.33	<0.001 ***	0.82
Exclusive	3.95	1.32	5.40	1.50	3.75	0.001 **	0.65
**During meal**							
Riceball (taste): salty	5.90	1.77	5.95	1.54	0.11	0.913	0.03
Riceball (texture): compact	5.95	2.01	6.80	0.95	1.82	0.084	0.39
Salad (texture): crunchy	7.35	0.99	7.40	1.39	0.86	0.878	0.19
Salad (color): colorful	6.20	1.99	7.10	1.48	2.85	0.010 *	0.55
Croquette batter (texture): soggy	5.30	1.87	5.45	1.82	0.29	0.772	0.07
Croquette ingredients (texture): dry	3.00	1.49	2.95	0.83	0.18	0.863	0.04
Corn soup (heat): hot	7.60	0.88	6.45	1.90	2.47	0.023 *	0.49
Corn soup (taste): natural sweetness of corn	6.40	1.60	6.70	1.78	0.78	0.445	0.18
**After meal**							
Liked	6.50	1.05	7.10	0.91	2.11	0.049 *	0.44
Contented	6.00	1.72	7.20	0.83	3.15	0.005 **	0.59
Want to eat it again	5.80	1.24	6.75	1.16	2.97	0.008 **	0.56
Relaxed	5.80	1.54	6.90	1.33	2.82	0.011 *	0.54
Was familiar	7.20	1.24	7.15	1.46	0.12	0.902	0.03

Notes: plastic, plastic tableware condition; natural, natural wooden tableware condition. Small, medium, and large effect sizes of *r* are 0.10, 0.30, and 0.50, respectively, *** *p* < 0.001 ** *p* < 0.01 * *p* < 0.05.

## Data Availability

The data presented in this study are available on request from the corresponding author.
